# The Determinants of Living with Long-Term Conditions: An International Cross-Sectional Study

**DOI:** 10.3390/ijerph181910381

**Published:** 2021-10-02

**Authors:** Silvia Corchon, Carmen Rodríguez-Blázquez, Alfonso Meneses, Marta Aranda-Gallardo, Lorena López, Maria Eugenia Ursúa, Maria Victoria Navarta-Sanchez, Mari Carmen Portillo, Leire Ambrosio

**Affiliations:** 1 Nursing Department, University of Valencia, 46010 Valencia, Spain; Silvia.Corchon@uv.es; 2 National Centre of Epidemiology and CIBERNED, Carlos III Institute of Health, 28029 Madrid, Spain; crodb@isciii.es; 3 Faculty of Nursing, Physiotherapy and Podiatry, University Complutense of Madrid, 28040 Madrid, Spain; ameneses@enf.ucm.es; 4 Department of Internal Medicine, Costa del Sol Hospital, Marbella, 29603 Malaga, Spain; maranda@hcs.es; 5 Madrid Healthcare System, 28823 Madrid, Spain; lorenabarbara1805@gmail.com; 6 Navarra Healthcare System, 31008 Navarra, Spain; eugenia.ursua.sesma@navarra.es; 7 Faculty of Medicine, Autonomous University of Madrid, 28029 Madrid, Spain; maria.navarta@uam.es; 8 NIHR ARC Wessex, Faculty of Health Sciences, University of Southampton, Southampton SO17 1BJ, UK; M.C.Portillo-Vega@soton.ac.uk

**Keywords:** chronic disease, determinants, person-centred care, cross-sectional studies

## Abstract

It is essential that healthcare and social professionals understand the daily lives of people with chronic diseases, and the variables that influence them. The aim of this study was to identify the determinants influencing the process of living with long-term conditions. To investigate this, an observational, international, cross-sectional study was carried out. A consecutive sample of 1788 Spanish-speaking population living with chronic obstructive pulmonary disease, chronic heart failure and type 2 diabetes mellitus were included. Descriptive statistics and multiple linear regression models were performed. The linear regression models identified that social support (β = 0.39, *p* < 0.001) and the satisfaction with life (β = 0.37, *p* < 0.001) were the main determinants in the process of living with a long-term condition (49% of the variance). Age (β = −0.08, *p* = 0.01) and disease duration (β = 0.07, *p* = 0.01) were determinants only in the chronic heart failure subgroup, and country was significant in the chronic obstructive pulmonary disease subgroup (β = −0.15, *p* = 0.002). Satisfaction with life and social support were key determinants influencing the process of living with long-term conditions. As such, those aspects should be included in the design of interventions focused on the achievement of a positive living in people with long-term conditions.

## 1. Introduction

Changes in life expectancy, demographics, lifestyles, healthcare, and social factors over the last century have led to a significant increase in chronic diseases or long-term conditions (LTCs) worldwide [1]. LTCs constitute one of the greatest challenges for healthcare and social systems, and are currently the leading cause of disability, morbidity, and costs [1]. Moreover, the projections of global mortality and the burden of diseases in the coming years estimate that LTCs will account for approximately three-quarters of all deaths globally in 2030, with huge socioeconomic impacts due to the exorbitant costs of often lengthy and expensive treatments [1,2]. Therefore, there is a growing need for the development of well-coordinated and cost-effective long-term care policies to address the consequences of this situation [3].

Among LTCs, chronic heart failure (HF), chronic obstructive pulmonary disease (COPD) and type 2 diabetes mellitus (T2DM) are the three most prevalent chronic conditions, constituting the principal causes of death worldwide [1,4]. HF is leading the LTC, with a prevalence of 10% in individuals older than 70 years of age [1,4,5]. COPD has become the third leading cause of death worldwide, with a prevalence of 251 million cases according to the Global Burden of Disease Study [4,6,7]. T2DM ranks seventh among the principal causes of death and its incidence has been estimated to increase to 693 million by 2045 [8].

In addition to this, it is important to consider that these conditions progressively worsen over time [2,6]. This leads people to experience an intensification of symptoms and limitations, which, in turn, affects their daily living, quality of life, and satisfaction with life [9,10]. When living with LTCs such as COPD, HF, and T2DM, people must adapt their daily routines and implement multiple adjustment behaviours [11,12,13]. The desired outcomes for these people are related to maintaining or improving their functional status, social life, and quality of life [9,11,12]. To achieve these goals, it could be helpful to understand how a person lives with an LTC and the determinants that could impact on this process. According to the World Health Organization [14], social determinants of health are defined as “the conditions in which people are born, grow, work, live and the set of forces and systems shaping the conditions of daily life”. In this sense, determinants encompass a wide array of variables that include social factors and similar broadly defined factors [15]. Living with LTCs is understood as a complex process that also includes internal processes [16]. It is not a static and linear process as people could shift through its different attributes moving from negative to positive living and vice versa [16].

It is essential that healthcare and social professionals understand the daily lives of people with LTCs and the variables that influence them. This understanding will generate the required knowledge to provide comprehensive, individualized, and person-centred care for those living with LTCs [17,18]. Until now, recent research has studied the variables related to a better quality of life or satisfaction with life in people living with LTCs [19,20,21,22]. These variables include age, gender, marital status, educational level [20,21], disease duration, symptom management, multimorbidity [19,22], social support [23,24], and satisfaction with life [25]. Based on this, to our knowledge, quality of life and satisfaction with life are two main consequences of the complex process of living with LTCs [16]. In this sense, the determinants of quality of life or satisfaction with life should not be generalized to those influencing the process of living with LTCs. Consequently, identification of the determinants of the process of living with LTCs from a comprehensive view and from the person’s perspective represents an important gap in the literature.

The aim of this study was to identify the determinants influencing the process of living with LTCs, such as COPD, HF, and T2DM.

## 2. Materials and Methods

### 2.1. Design

An observational, international, and cross-sectional study [26] was carried out.

### 2.2. Setting and Participants

This was a multicentre study that included public and private hospitals, primary and secondary specialized units, and patient associations or community groups of Spain and Colombia. The sample for this study was composed of outpatients who met the following inclusion and exclusion criteria (Table 1):

Consecutive case sampling was performed [27,28].

To obtain the convenience sample of people living with COPD, HF, or T2DM from both countries, a minimum sample size of 260 people per pathology and country was established [29]. This sample size was calculated for a factory analysis process as part of the LW-CI scale validation [30,31]. In this sense, a total of 780 people per country were established, with a consecutive total sample size of 1560 people living with COPD, HF, and T2DM.

### 2.3. Study Variables and Instruments

The dependent variable of this study was living with LTCs. The independent variables of the study included sociodemographic variables, namely country, gender, age, marital status, educational level, disease duration, employment situation, social support, satisfaction with life, and severity of the illness perceived by the person. The Spanish validated version of the following instruments was used to measure those variables.

The Living with Chronic Illness Scale (LW-CI scale) [32] is a self-reported measuring scale to evaluate the complex process of living with an LTC through 26 items grouped into the domains of acceptance (4 items), coping (7 items), self-management (4 items), integration (5 items), and adjustment (6 items) [32]. All items are answered using a 5-point Likert scale from never or nothing (0) to always or a lot (4), except for the domain acceptance, which is reversely scored from never or nothing (4) to always or a lot (0). In this way, the LW-CI scale has total score value from 0 points, indicating negative living with the LTC, to 104 points, reflecting positive living with the LTC [32].

The Duke-UNC Functional Social Support questionnaire (DUFSS) [33,34] is an 11-item scale that was used to evaluate perceived social support of the person when living with the LTC including areas, such as confidant, affective, and instrumental support [33,34]. Each item is scored from 1 (much less than I would like) to 5 (as much as I would like). The total score ranged from 11 (the lowest level of support) to 55 (the highest level of perceived social support) [33,34].

A modified version of the Satisfaction with Life Scale (SLS-6) [35] was used to evaluate satisfaction with life during the process of living with an LTC. The SLS-6 is a 6-item scale related to physical area, psychological wellbeing, social relations, leisure, and financial situation. Each item is scored on a Likert scale from 0 (totally unsatisfied with life) to 10 (totally satisfied with life) [35].

The Patients-based Global Impression of Severity Scale (PGIS) [36,37] was used to evaluate the self-perception of the disease severity. This is rated on a 6-point Likert scale, using a range of responses from 0 (not ill at all) to 5 (extremely ill) [36,37].

### 2.4. Ethical Issues

The study was approved by the Ethics Committees of the participant hospitals and the participating universities in Spain (reference number 2017.099) and Colombia (reference number 013). The study followed the Declaration of Helsinki and the standard operating procedures that guaranteed compliance with good clinical practice. After receiving pertinent oral and written information and before inclusion in the study, all participants signed informed consent forms.

### 2.5. Data Collection

As explained previously in publications regarding LW-CI scale validation in different LTCs [30,31] for data collection, the principal researcher developed a detailed protocol indicating the steps of the study with the aim of reducing potential errors and heterogeneity in the process. This protocol was then sent to the responsible researcher of the centre that was undertaking the data collection. Moreover, a meeting was organised to provide researchers an opportunity to ask questions and clarify doubts. Thus, before starting data collection, the principal investigator ensured that all researchers involved in this process understood the established steps [30,31].

All the researchers and centres of the study followed the detailed protocol for data collection: (1) healthcare professionals (nurses and physicians) approached all the potential participants giving initial oral information about the study, (2) interested people received an invitation letter and the participant information sheet with detailed written information, and (3) participants completed the questionnaires during the routine clinical visits. Although all the scales were self-reported, the researcher was available to resolve any doubts that could arise during their completion [30,31].

### 2.6. Statistical Analysis

Descriptive statistics were applied to characterize the participants’ sociodemographic and disease-related data. Multiple linear regression models were performed using the LW-CI scale total score and the total score of the scale for each LTC (LW-CI-HF scale [31], LW-CI-COPD scale, and LW-CI-T2DM scale) as the dependent variable. Independent variables included sociodemographic aspects, social support (DUFSS), satisfaction with life (SLS-6), and persons’ perception of the severity of the illness (PGIS).

Assumptions for the linear regression model (normality, homoscedasticity, independence of errors, and absence of multicollinearity) were assessed; therefore, some variables, such as age at disease onset, were excluded due to collinearity.

The enter method was used for the regression models to simultaneously assess the effect of each explanatory variable in each model, considering that multicollinearity was previously discarded. *p*-values of 0.05 or less were considered statistically significant. All calculations were performed using IBM SPSS 25.0 statistical software.

## 3. Results

A total sample of 1788 people living with LTCs from Spain and Colombia were included in this study. As shown in Table 2, 52.1% of participants from the total sample were men. The mean age of the sample was 68.9 years (standard deviation, SD 12.3), and most participants were married (55.4%), retired (35.3%), and had a primary or basic educational level (62.1%). The mean disease duration was 8.7 (SD 7.9) years.

The sociodemographic characteristics of the sample per LTC and historical data of each condition are presented in Supplementary Material S1.

Regarding linear regression models, using the LW-CI total scale score for the whole sample, the main determinants were the DUFSS (standardized beta, β = 0.39, *p* < 0.001) and SLS-6 (β = 0.37, *p* < 0.001) scales (see Table 3). This model accounted for 49% of the variance.

DUFSS (β = 0.33, *p* < 0.001) and SLS-6 (β = 0.45, *p* < 0.001) together with age (β = −0.08, *p* = 0.01), and disease duration (β = 0.07, *p* = 0.01) were also the main determinants for living with HF, accounting for 56% of the variance (see Table 3).

The LW-CI-T2DM results also showed that DUFSS (β = 0.39, *p* < 0.001) and SLS-6 (β = 0.31, *p* < 0.001) were the main determinants in a model accounting for 41% of the variance (Table 3).

As shown in Table 4, for LW-CI-COPD, the country was a significant variable (β = −0.15, *p* = 0.002). Thus, separate models were performed for Spain and Colombia.

For Spain, only marital status was significant (β = 0.38, *p* < 0.01), accounting for 1% of the variance. For Colombia, DUFSS (β = 0.51, *p* < 0.001), SLS-6 (β = 0.26, *p* < 0.001) and marital status (β = 0.10, *p* < 0.01) were significantly associated with LW-CI-COPD, accounting for 52% of the variance (Table 4).

## 4. Discussion

To our knowledge, this is the first study that has focused on the determinants in the process of living with LTCs from a comprehensive perspective.

Data were captured on a wide sample of people with LTCs from two different Spanish-speaking countries: Spain and Colombia. This allowed an understanding of the process of living with LTCs in people with different backgrounds and cultural contexts. Specifically, it explored the determinants associated with living with COPD, HF, and T2DM, pathologies with high prevalence worldwide and great impact on people’s life due to their symptoms and potential exacerbations [4]. Therefore, the sample size and its heterogeneity regarding the most prevalent conditions currently as well as the sociodemographic characteristics support its generalization to people living with these LTCs.

Regarding the variables that influenced the process of living with LTCs, the results indicate that the perceived social support and satisfaction with life were key aspects for people in this study. To our knowledge, social support includes health professionals’ support as well as family, partners, friends, community groups, and voluntary or charity organisations [11,12,13,38]. This support contributes to relieving people of stress, improving their acceptance, coping, adjustment to the disease and reinforcing self-care and psychological well-being [11,12,13,38]. Our results are congruent with previous studies investigating people living with different LTCs [38,39,40,41], stressing that social support was related to better reported general and emotional health in people as well as well-being and quality of life. For example, our study mirrors previous studies that investigated people living with Parkinson’s disease [42], wherein social support was strongly correlated with the process of living with the illness. This means that social support is a significant and independent influence on the process of living with different LTCs, such as Parkinson’s disease, COPD, HF, and T2DM. This supports the fact that there are important parallels between different LTCs that could result in common care pathways and interventions. Consequently, it is important to develop interventions to foster person’s living with LTCs, promoting better quality of life, psychosocial wellbeing, and health-related outcomes [16]. Other studies that investigated a COPD population have identified associations between the social support received through a comprehensive intervention and the perceived symptoms and person’s quality of life [39,43,44,45]. Regarding people living with HF, some authors [24] highlighted that psychological health and social relationships were strongly related to the daily living of people, whereas physical health presented a slight association with living with HF. However, these results should be taken with caution as it was a qualitative study undertaken in a non-generalizable HF population. Other studies [11,12,46] conducted in a population with T2DM also stressed the crucial role of social support, especially from the family, in people’s experience with the illness. Similar results were drawn in studies conducted with people living with neurological conditions, such as chronic stroke [47] and Parkinson’s disease [Ambrosio 2019] where the positive effect of social support programs had on the persons’ mental health and well-being is highlighted. Regarding satisfaction with life, previous studies also found that a more satisfactory life was related to better daily living with LTCs, such as HF [24,48], COPD [25], and T2DM [49]. Therefore, it could be highlighted that social support and satisfaction with life seem to be key factors in the process of living with LTCs. These results are paramount for the development of mental health programs and person-centred pathways to promote positive living with LTCs and maximise quality of life, wellbeing, and health-related outcomes such as satisfaction with life.

In line with previous publications that have found strong associations of emotional and social support and participants’ self-reported health, wellbeing, and quality of life [40,41], findings emerged in this study that revealed some differences in the determinants of positive living depending on the pathology and people’s characteristics. For example, according to the results of this study, age and the duration of the illness are determinants in people living with HF. This is coherent with existing literature, showing that people living with HF have an important reduction of their quality of life due to the disease progression, particularly in patients with HF in New York Heart Association Classification classes II (mild symptoms and slight limitation during ordinary activity) and III (marked limitation in activity due to symptoms, comfortable only at rest) [50]. This variation has not been identified in people with other LTCs such as T2DM and COPD, so patients with HF may need a more extensive follow-up as the disease progresses [50].

Treatment and its characteristics did not seem to be determinants in the process of living with the illness in any of the three pathologies (COPD, T2DM, and HF) examined. In other words, it appears that independent of the received treatment, the person could experience negative or positive living with the LTC. Similar results have been identified in previous studies of people living with other LTCs, such as Parkinson’s disease or chronic stroke [47]. Therefore, it could be concluded that in these LTCs (COPD, T2DM, HF, Parkinson’s disease, or chronic stroke), living with the pathology is a process intrinsically related to the individual characteristics rather than to the illness and the treatment itself. In fact, of the independent variables introduced in this study (age, gender, educational level, employment situation, disease duration, and person’s perception of LTC severity), only social support and satisfaction with life seemed to be determinants in the process of living with LTCs. However, regarding the treatment, it is important to highlight the particular risk of medical errors. These errors, which in many cases occur in domestic settings [51], may have a dramatic impact on people with LTCs, and may potentially be responsible for countless adverse effects and even the death of these persons [52]. Therefore, these issues and the potential impact on the well-being and quality of life of people with different LTCs should be further explored in future research studies.

Regarding possible differences between countries, this was only noted in people living with COPD. For the Colombian population, social support, satisfaction with life, and marital status were determinant factors in the process of living living with the condition. However, in the Spanish population, only marital status was a determinant. This unexpected result could be explained by differences in the characteristics of people, or in the care provided to this specific population in Latin America and European countries. To our understanding, most knowledge of COPD has been based on research carried out in Europe or North America and there is a gap of information in people from Latin America about the prevalence, person’s characteristics, and changes in lung function over time [53,54]. Nevertheless, the model for the Spanish population accounted for only 1% of the variance. Therefore, it seems that in Spanish people living with COPD, there are other variables that could be determinant and have not been taken into account in this study. However, due to the lack of previous studies focused on these identified potential differences, it is not easy to provide an explanation for this finding, and more research is needed to explore these potential differences in detail.

This study has some limitations that should be taken into account. Although the variance explained by the models is relatively high, there could be other determinant variables in the process of living with LTCs that have not been included in this study (such as comorbidity or multimorbidity) [21]. This study included people from two different countries and with three specific LTCs. As such, the determinants in the process of living with LTCs could vary in people with other pathologies or living in different contexts. Therefore, caution is needed to extrapolate these results to other prototypical LTCs and other Spanish-speaking countries with different cultures. Moreover, this was a cross-sectional study, so it is difficult to establish causal relationships. Therefore, further analytic studies that include people from different countries and with other LTCs are highly recommended.

This study also presented several strengths that should be highlighted: the large sample size; a heterogeneous representation of people living with different prototypical and highly prevalent LTCs (T2DM, COPD and HF); and participants from different settings, such as health care centres and community centres, as well as two different Spanish-speaking countries. Therefore, this study could contribute to knowledge across countries to identify synergies between professionals of different disciplines and sectors to address the process of living with LTCs from a person-centred perspective.

## 5. Conclusions

In conclusion, this study has allowed us to identify the variables associated with the process of living with LTCs. Here, we highlighted the necessity of a comprehensive approach involving health and social care that focuses on the person and not on the disease. Satisfaction with life and social support have been identified as determinants for people living with LTCs. Therefore, social support assessment should be addressed in the health care and social system. This work has led to a new understanding of essential elements that self-management programs and health and social care interventions need to target for a more positive living with LTCs. In this sense, this research has highlighted the necessity of increasing the focus on capturing the determinants that are particularly important for people. Understanding these determinants could enhance their health outcomes, quality of life, and process of living with LTCs. This study provides valuable information for the development of effective long-term care policies for the management of LTCs, one of the principal challenges faced by modern society.

## Figures and Tables

**Table 1:** Inclusion and exclusion criteria.

**Inclusion Criteria**	People diagnosed with COPD, HF or T2DM by a General Practitioner, pneumologist, endocrinologist or cardiologist
	Native Spanish-speaking population
	People who were able to read and understand questionnaires properly
	People who were able to provide informed consent
**Exclusion Criteria**	People with cognitive deterioration, mental disorders or pharmacological side-effects that could potentially disrupt the assessment

COPD: chronic obstructive pulmonary disease; HF: heart failure; T2DM: type 2 diabetes mellitus.

**Table 2:** Sociodemographic and disease characteristics of the total sample.

Variables	Categories	n (%)
**Gender**	Men	937 (52.1)
	Women	860 (47.9)
**Marital status**	Single	217 (12.1)
	Married	994 (55.4)
	Widow	416 (23.1)
	Other	167 (9.3)
**Employment situation**	Employee	282 (15.7)
	Housekeeper	569 (31.7)
	Retired	635 (35.3)
	Other	310 (17.3)
**Educational level**	Primary or basic	1113 (62.1)
	Secondary level	442 (24.7)
	University	203 (11.3)
	Other	35 (2.0)
	**Range (years)**	**Mean (SD)**
**Age**	20–98	68.9 (12.3)
**Age at LTC onset**	3–91	60.1 (12.8)
**Duration of the LTC**	0–67	8.7 (7.9)

LTC: long-term condition.

**Table 3:** Multiple linear regression models.

Independent Variables *	LW-CI Total Sample (n = 1788)		LW-CI-HF (n = 603)		LW-CI-T2DM (n = 582)		LW-CI-COPD (n = 612)	
	Standardized Beta	*p*	Standardized Beta	*p*	Standardized Beta	*p*	Standardized Beta	*p*
***(Constant)***	*5.08*	*<0.001*	*2.25*	*0.02*	*2.36*	*0.02*	*3.34*	*0.00*
**Age**	−0.04	0.06	−0.08	0.01	0.03	0.45	0.03	0.57
**Country**	0.04	0.07	0.06	0.08	0.05	0.24	−0.02	0.002
**Marital status**	0.03	0.11	−0.01	0.98	0.47	0.17	0.11	0.003
**Disease duration**	0.04	0.03	0.07	0.01	0.05	0.19	−0.29	0.47
**DUFSS**	0.39	<0.001	0.33	<0.001	0.39	<0.001	0.53	<0.001
**SLS-6**	0.37	<0.001	0.45	<0.001	0.31	<0.001	0.28	<0.001
**Adj R-squared**	0.49		0.56		0.41		0.48	

LW-CI: Living with Chronic Illness scale; LW-CI-HF: Living with Chronic Illness—heart failure scale; LW-CI-T2DM: Living with Chronic Illness—type 2 diabetes mellitus; LW-CI-COPD: Living with Chronic Illness—chronic obstructive pulmonary disease; DUFSS: Duke-UNC Functional Social Support Questionnaire; SLS-6: Satisfaction with Life Scale. * Independent variables with at least one significant result (*p* < 0.05). Other variables included in the models were: gender, educational level, employment situation, and Patient-based Global Impression of Severity Scale (PGIS).

**Table 4:** Multiple linear regression models of LW-CI-COPD by country.

Independent Variables *	Spain (n = 279)		Colombia (n = 333)	
	Standardized Beta	*p*	Standardized Beta	*p*
**(Constant)**	1.20	0.24	0.69	0.49
**Marital status**	0.38	0.10	0.10	0.01
**DUFSS**	0.29	0.10	0.51	<0.001
**SLS-6**	0.09	0.58	0.26	<0.001
**Adj R-squared**	0.09		0.52	

LW-CI-COPD: Living with Chronic Illness—chronic obstructive pulmonary disease; DUFSS: Duke-UNC Functional Social Support scale; SLS-6: Satisfaction with life scale. * Other variables included in the models: age, gender, educational level, employment situation, disease duration, and Patient-based Global Impression of Severity Scale (PGIS).

## Data Availability

The data presented in this study are available on request from the corresponding author. The data are not publicly available due to confidentiality of participants.

## Supplementary Materials

The following are available online at https://www.mdpi.com/article/10.3390/ijerph181910381/s1, Table S1: Sociodemographic characteristics of the sample per long-term condition and historical data of the condition.
