# Supplementary material for: The Determinants of Living with Long-Term Conditions: An International Cross-Sectional Study
Source: Int J Environ Res Public Health. 2021 Oct 2;18(19):10381. doi: 10.3390/ijerph181910381 (PMC8508439; doi:10.3390/ijerph181910381)
Supplement: Supplementary file 1 [file ijerph-18-10381-s001.zip › ijerph-1377247-supplementary.pdf]

Table S1. Sociodemographic characteristics of the sample per long-term condition and historical data of the condition.

Variables	Categories	LTCs		
		COPD	CHF	T2DM
		n (%)	n (%)	n (%)
**Gender**	Men	341 (55.7)	320 (53.1)	276 (47.42)
	Women	271 (44.3)	283 (46.9)	306 (52.6)
**Marital status**	Single	80 (13.1)	50 (8.3)	87 (15.0)
	Married	303 (49.5)	358 (59.4)	333 (57.5)
	Widow	178 (29.1)	145 (24)	93 (16.1)
	Other	51 (8.3)	50 (8.3)	66 (11.4)
**Employment situation**	Employee	51 (8.3)	57 (9.5)	174 (29.9)
	Housekeeper	211 (34.5)	199 (33)	159 (27.4)
	Retired	214 (35.0)	300 (49.8)	121 (20.8)
	Other	136 (22.2)	47 (7.8)	127 (21.9)
**Educational level**	Primary or basic	367 (60.2)	396 (65.7)	350 (60.3)
	Secondary level	165 (27.0)	143 (23.7)	134 (23.1)
	University	66 (10.8)	52 (8.6)	85 (14.7)
	Other	12 (2.0)	12 (2)	11 (1.9)
	**Range (years)**	**Mean (Standard Deviation)**		
**Age**		70.62 (11.96)	71.7 (11.4)	64.15 (12.18)
**Age at LTC onset**		62.26 (11.80)	63.9 (12)	
**Duration of the LTC**		6.91 (6.0)	7.8 (7.4)	10.25 (9.51)

LTCs: Long-term conditions. COPD: chronic obstructive pulmonary disease; HF: chronic heart failure; T2DM: type 2 diabetes mellitus.
